# Interdependency of Reactive Oxygen Species generating and scavenging system in salt sensitive and salt tolerant cultivars of rice

**DOI:** 10.1186/s12870-016-0824-2

**Published:** 2016-06-10

**Authors:** Navdeep Kaur, Manish Dhawan, Isha Sharma, Pratap Kumar Pati

**Affiliations:** Department of Biotechnology, Guru Nanak Dev University, Amritsar, 143005 Punjab India; Department of Oral biology, Augusta University, Augusta, GA USA

**Keywords:** Rice, Salinity, Reactive oxygen species (ROS), NADPH oxidase, Hydrogen peroxide (H_2_O_2_), Antioxidant enzymes

## Abstract

**Background:**

Salinity stress is a major constrain in the global rice production and hence serious efforts are being undertaken towards deciphering its remedial strategies. The comparative analysis of differential response of salt sensitive and salt tolerant lines is a judicious approach to obtain essential clues towards understanding the acquisition of salinity tolerance in rice plants. However, adaptation to salt stress is a fairly complex process and operates through different mechanisms. Among various mechanisms involved, the reactive oxygen species mediated salinity tolerance is believed to be critical as it evokes cascade of responses related to stress tolerance. In this background, the present paper for the first time evaluates the ROS generating and the scavenging system in tandem in both salt sensitive and salt tolerant cultivars of rice for getting better insight into salinity stress adaptation.

**Results:**

Comparative analysis of ROS indicates the higher level of hydrogen peroxide (H_2_O_2_) and lower level of superoxide ions (O^2-^) in the salt tolerant as compared to salt sensitive cultivars. Specific activity of ROS generating enzyme, NADPH oxidase was also found to be more in the tolerant cultivars. Further, activities of various enzymes involved in enzymatic and non enzymatic antioxidant defence system were mostly higher in tolerant cultivars. The transcript level analysis of antioxidant enzymes were in alignment with the enzymatic activity. Other stress markers like proline were observed to be higher in tolerant varieties whereas, the level of malondialdehyde (MDA) equivalents and chlorophyll content were estimated to be more in sensitive.

**Conclusion:**

The present study showed significant differences in the level of ROS production and antioxidant enzymes activities among sensitive and tolerant cultivars, suggesting their possible role in providing natural salt tolerance to selected cultivars of rice. Our study demonstrates that the cellular machinery for ROS production and scavenging system works in an interdependent manner to offer better salt stress adaptation in rice. The present work further highlights that the elevated level of H_2_O_2_ which is considered as a key determinant for conferring salt stress tolerance to rice might have originated through an alternative route of photocatalytic activity of chlorophyll.

## Background

Rice is an important cereal crop that has the potential to provide food security to the increasing world population. Out of the total global agricultural land, 150 million hectares is estimated to be under rice cultivation that leads to an annual production averaging to 500 million metric tons [1]. But a large amount of the rice biomass is not harvested under field conditions due to sensitivity of the crop to various abiotic stresses like drought, salinity, low temperature, heat shock [2, 3]. Among these stresses, salinity is a major constrain to rice production worldwide which adversely affect its growth and development at the molecular, biochemical and physiological level [4, 5]. It affects more than 20 % of total cultivated land worldwide that results in US$12 billion loss in global agricultural production and this loss is further increasing each year [5, 6]. As in the present scenario of global climate change, the level of land salinization is expected to increase and hence there is an immediate world-wide concern for development of better salt tolerant cultivars for future food security. For achieving this objective, a thorough comparative analysis of salt sensitive and tolerant cultivars coupled with increased understanding of the underlying mechanism involved in salt stress adaptation is much warranted.

Reactive oxygen species (ROS) generation, signalling and detoxification are vital components of salt stress adaptation mechanisms and a priority area of research worldwide for the accomplishment of better growth and yield of a crop under salt affected areas [7]. ROS such as superoxide radical (O_2_^-^), hydroxyl radical (OH^-^) and hydrogen peroxide (H_2_O_2_) are produced in a low concentration as a result of normal cellular metabolism in plants and plays important role in growth, development and in adaptation to stress [8]. But, when a plant is subjected to stress, the perturbation of ROS homeostasis takes place which triggers multiple signalling responses involved in critical functions of plants [9]. Hence, a fine tuned balance between ROS production and scavenging is crucial for the plant survival under unfavourable conditions.

NADPH oxidases are one of the major enzyme systems involved in production of ROS in the apoplastic space under stress [10]. They catalyze the transfer of electons from NADPH to molecular oxygen (O_2_) for the generation of free radical O^2-^ [11]. NADPH oxidases hold a distinct position among the various ROS generating enzyme systems in plants because of their role in different signalling pathways involved in plant growth, development and stress tolerance [12]. Superoxide ions produced as a result of enzymatic activity of NADPH oxidase is converted into H_2_O_2_ via superoxide dismutase (SOD) and it diffuses to adjacent cellular components where it acts as a signalling molecule that activates various stress responsive pathways. This indicates that the coordination of ROS generating and scavenging system might play a critical role in salt stress adaptation mechanism. However, the exact role of NADPH oxidase in conferring salt stress adaptation is not yet properly understood.

Earlier studies conducted on salt tolerant cultivar Pokali and sensitive cultivar IR64 under the effect of salt treatment has indicated a definitive link between scavenging system and salinity tolerance [13]. However, to best of our knowledge no work has been initiated to compare the ROS generating and scavenging system together in salt sensitive and tolerant cultivars. Therefore, in the present study, a comparative study of ROS dynamics was conducted in salt tolerant (Luna Sankhi and Luna Suvarna) and salt sensitive (IR64 and Pusa Basmati-1) cultivars of rice. The information gained on real time situation on this critical stress alleviating ROS pathway will facilitate in developing cultivars with better salt stress adaptation. Further, this work adds to the existing knowledge of our understanding on the complex physiological, biochemical and genetic mechanisms involved in salt stress adaptation.

## Results

### Histochemical analysis of ROS

**Fig. 1:**
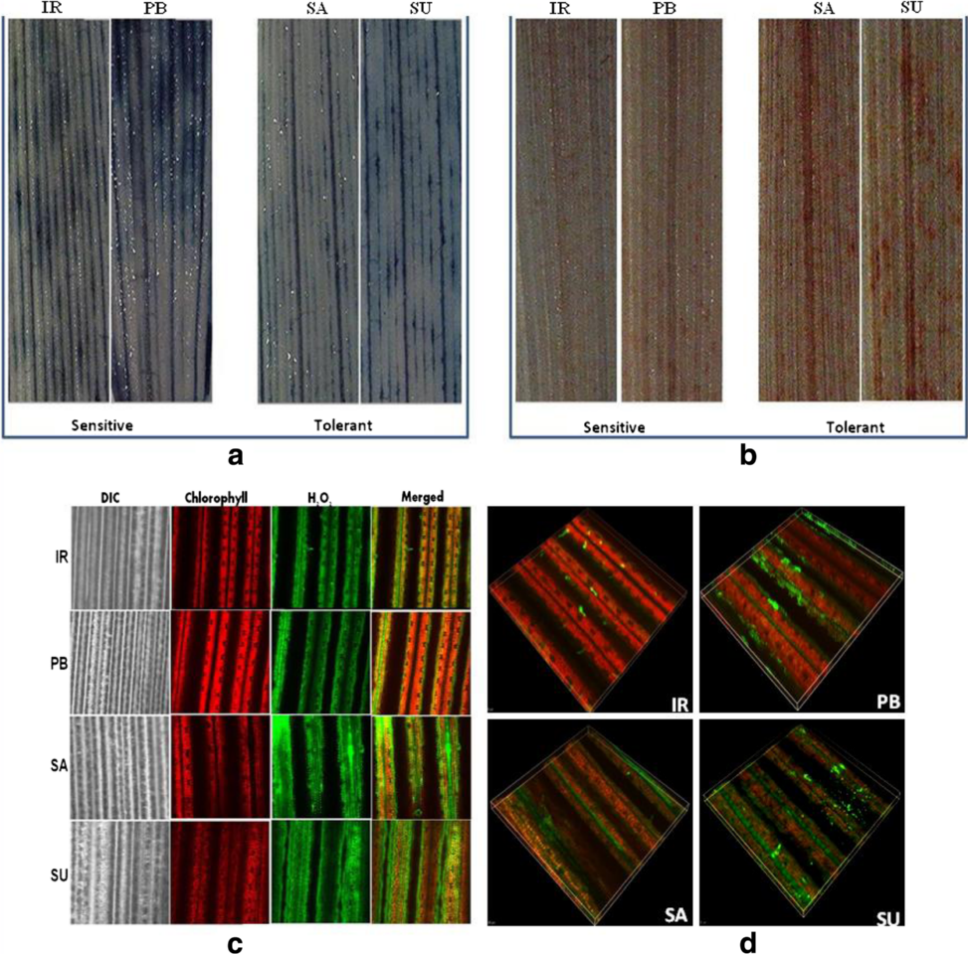
O2^-^ and H_2_O_2_ accumulation in leaves of different cultivars of rice. Leaves were excised at the base of stems and were supplied with different stains. (**a**) NBT staining (**b**) DAB staining (**c**) Confocal images of H_2_DCFDA staining (**d**) 3D-view of confocal H_2_DCFDA staining images (IR: IR64, PB: Pusa Basmati-1, SA: Luna Sankhi, SU: Luna Suvarna)

The level of different types of ROS among salt sensitive and tolerant cultivars was monitored histochemically using three different dyes (Fig. 1). Nitrozolium blue (NBT) assay showed higher accumulation of dark blue coloured pigment that estimates the higher level of O^2-^ in the rice cultivars IR64 and Pusa Basmati-1. On the contrary to this, 3, 3-diaminobenzidine (DAB) showed higher level of H_2_O_2_ that was assessed through the intensity of dark brown coloured spots in tolerant cultivars (Luna Sankhi and Luna Suvarna). Similarly, staining using 2', 7’-dichlorodihydrofluorescein diacetate (H_2_DCFDA) which binds to ROS (predominantly H_2_O_2_) yielded a higher intensity of green fluorescence in tolerant cultivars as compared to the sensitive cultivars.

### NADPH oxidase activity assay

**Fig. 2:**
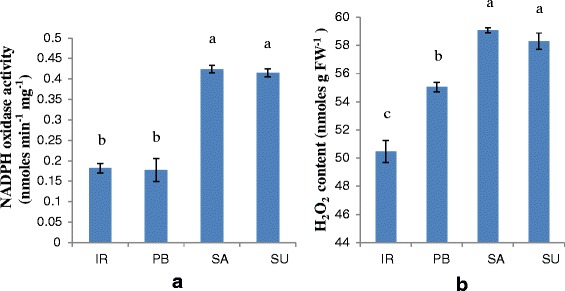
Comparative analysis of (**a**) NADPH oxidase activity and (**b**) H_2_O_2_ content of 14 days old seedlings of different rice cultivars. (IR: IR64, PB: Pusa Basmati-1, SA: Luna Sankhi, SU: Luna Suvarna). Bars represent mean ± SE (*n* = 3). Different letters (a, b, c) within cultivars are significantly different (Tukey LSD, *p* ≤ 0.05)

The quantitative analysis of ROS generating NADPH oxidase enzyme activity showed significant difference among four cultivars of rice (Fig. 2). The enzymatic activity (nmoles min^-1^ mg^-1^) was found to be significantly high in Luna Sankhi (0.424 ± 0.009) followed by Luna Suvarna 0.415 ± 0.010 than the sensitive cultivar IR64 (0.182 ± 0.011) and Pusa Basmati-1 (0.177 ± 0.028).

### H_2_O_2_ content estimation

The estimation of aqueous H_2_O_2_ content in four varieties showed considerable difference as expected on the basis of histochemical assays (Fig. 2). The content of aqueous H_2_O_2_ (units nmoles g^-1^ FW) was found to be significantly more in tolerant cultivars Luna Sankhi (59.076 ± 0.187) and Luna Suvarna (58.296 ± 0.585) as compared to sensitive varieties IR64 (50.467 ± 0.76) and Pusa Basmati-1 (55.042 ± 0.340) which were at par with each other.

### Antioxidant enzyme activity

Significant differences in the specific activity of various antioxidant enzymes were observed in different cultivars (Table 1). The overall specific activity of all the antioxidant enzymes, except catalase (CAT) was found to be higher in tolerant cultivars. SOD that converts superoxide ions into hydrogen peroxide showed significantly higher specific activity (units mg protein^-1^) in Luna Sankhi (0.0661 ± 0.0033) and Luna Suvarna (0.0866 ± 0.0008) with respect to IR64 (0.0243 ± 0.0023) and Pusa Basmati-1 (0.0387 ± 0.0021). The specific activity of ascorbate peroxidase (APX) was augmented to a significantly higher in Luna Sankhi (26.21 ± 0.301) and Luna Suvarna (26.13 ± 0.230) as compared to IR64 (19.85 ± 0.563) and Pusa Basmati-1 (18.55 ± 0.518) but on the contrary the specific activity of CAT was more in sensitive cultivars IR64 (0.486 ± 0.012) and Pusa Basmati-1 (0.581 ± 0.003) in comparison to Luna Sankhi (0.277 ± 0.190) and Luna Suvarna (0.397 ± 0.013) (Table 1). The enzymes involved in the well known glutathione-ascorbate pathway also showed considerable difference in their specific activity among different cultivars. The specific activity of glutathione reductase (GR) was found to be higher in Luna Sankhi (5.98 ± 0.276) and Luna Suvarna (5.91 ± 0.072) than IR64 (3.58 ± 0.045) and Pusa Basmati-1 (4.12 ± 0.112) (Table 1). Similarly, guaiacol peroxidase enzyme (GPX) also showed similar type of trend with higher activity in Luna Sankhi (68.41 ± 3.45) and Luna Suvarna (75.51 ± 1.68) followed by IR64 (51.16 ± 1.65) and Pusa Basmati-1 (51.80 ± 0.40). Dehydroascorbate reductase (DHAR) and monodehydroascorbate reductase (MDHAR) activities were also found to be high in tolerant cultivars (Luna Sankhi and Luna Suvarna) in comparison to the sensitive ones (IR64 and Pusa Basmati-1) (Table 1).

**Table 1:** Comparative analysis of different antioxidant enzymes and ascorbate content in 14 days old seedlings of different rice cultivars

Cultivar	SOD (units mg protein^-1^)	APX (μmol min^-1^ mg protein^-1^)	CAT (μmol min^-1^ mg protein^-1^)	GPX (μmol min^-1^ mg protein^-1^)	GR (μmol min^-1^ mg protein^-1^)	DHAR (μmol min^-1^ mg protein^-1^)	MDHAR (μmol min-1 mg protein^-1^)	Reduced ascorbate/Oxidised ascorbate ratio
IR64	0.0243 ± 0.0023^d^	19.85 ± 0.563^b^	0.486 ± 0.012^a^	51.16 ± 1.65^b^	3.58 ± 0.045^b^	1.02 ± 0.016^b^	1.76 ± 0.068^b^	0.739 ± 0 .027^b^
PB	0.0387 ± 0.0021^c^	18.55 ± 0.518^b^	0.581 ± 0.003^a^	51.80 ± 0.40^b^	4.12 ± 0.112^b^	1.05 ± 0.014^b^	1.60 ± 0.063^b^	0.698 ± 0.011^b^
Luna Sankhi	0.0661 ± 0.0033^b^	26.21 ± 0.301^a^	0.277 ± 0.190^b^	68.41 ± 3.45^a^	5.98 ± 0.276^a^	1.43 ± 0.05^a^	2.24 ± 0.060^a^	0.884 ± 0.017^a^
Luna Suvarna	0.0866 ± 0.0008^a^	26.13 ± 0.230^a^	0.397 ± 0.013^b^	75.51 ± 1.68^a^	5.91 ± 0.072^a^	1.49 ± 0.05^a^	2.28 ± 0.075^a^	0.905 ± 0.033^a^

(*SOD* Superoxide Dismutase, *APX* Ascorbate Peroxidase, *CAT* Catalase, *GPX* Guaicol Peroxidase, *GR* Glutathione Reductase, *DHAR* Dehydroascorbate Reductase, *MDHAR* Monodehydroascorbate Reductase). Values represent mean ± SE (*n* = 3)

Different letters (^a^, ^b^, ^c^, ^d^) within cultivars are significantly different (Tukey LSD, *p* ≤ 0.05)

### Expression analysis of antioxidant enzymes

**Fig. 3:**
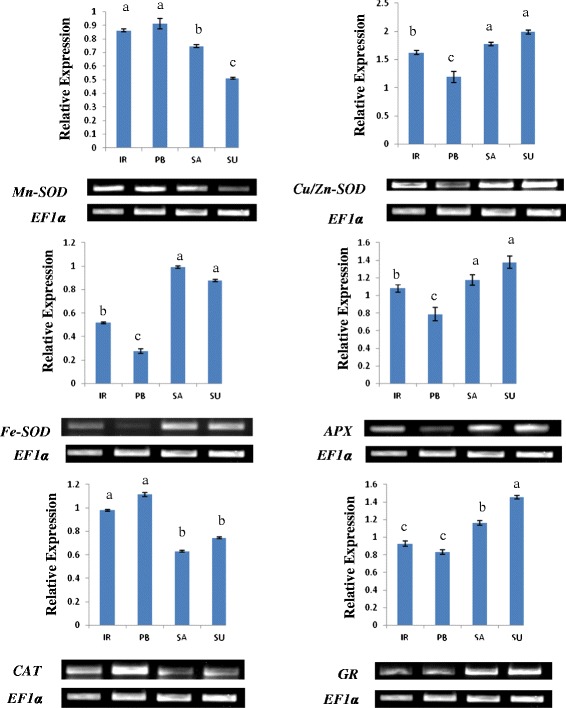
An ethidium bromide stained agarose gel harbouring products from reverse transcriptase-PCR of different key antioxidant genes of 14 days old seedlings of different rice cultivars. Gel shows the PCR products after 35 cycles of PCR and the results were first normalized to the housekeeping gene *EF1α.* Bars represent mean ± SE (*n* = 3). Different letters (a, b, c, d) within cultivars are significantly different (Tukey LSD, *p* ≤ 0.05). (IR: IR64, PB: Pusa Basmati-1, SA: Luna Sankhi, SU: Luna Suvarna)

**Fig. 4:**
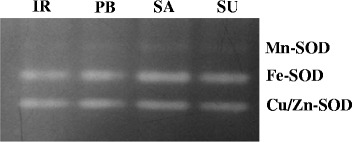
In-gel analysis of the pattern of SOD isoenzymes in two salt sensitive and two salt tolerant cultivars of rice (IR: IR64, PB: Pusa Basmati-1, SA: Luna Sankhi, SU: Luna Suvarna)

The semi-RT PCR analysis of various antioxidant genes also showed higher level of key antioxidant enzymes in tolerant cultivars except *CAT* and *Mn-SOD* at the transcript level (Fig. 3). Among the different isoforms of SOD, the transcript level of *Fe-SOD* and *Cu/Zn-SOD* showed much higher accumulation in Luna Sankhi and Luna Suvarna. These results were further confirmed at the protein level with in–gel SOD assay (Fig. 4). On the contrary, *Mn-SOD* showed lesser expression in tolerant cultivars when compared against the sensitive cultivars. The transcript level of *CAT* was also found to be more in the sensitive cultivars (IR64 and Pusa Basmati-1). However, *APX* and *GR* enzymes were found in the same type of trend at the transcript level as followed in their respective enzymatic activities. Their gene expression was also higher in Luna Sankhi and Luna Suvarna as compared to the sensitive ones (IR64 and Pusa Basmati-1).

### Ascorbate content

A notable difference in the reduced to oxidised ascorbate ratio was found in 4 cultivars of rice (Table 1). Tolerant cultivars Luna Suvarna and Luna Sankhi showed significantly higher ratio of (0.905 ± 0.033) and (0.884 ± 0.017), respectively in contrast to the sensitive cultivars IR64 (0.739 ± 0.027) and Pusa Basmati-1 (0.698 ± 0.011).

### Total protein, free proline and MDA content analysis

In this study, we estimated the total protein content in the four cultivars. The protein content (mg FW ^-1^) was found to be significantly high in tolerant cultivars Luna Sankhi (14.73 ± 0.275) and Luna Suvarna (15.22 ± 0.403) with respect to the sensitive cultivars IR64 (12.75 ± 0.275) and Pusa Basmati-1(12.85 ± 0.352) (Table 2). The significantly enhanced content of proline (μmoles g FW ^-1^) was found in Luna Sankhi (6.90 ± 0.0513) and Luna Suvarna (6.78 ± 0.2450) against the sensitive cultivar IR64 (4.29 ± 0.0177) and Pusa Basmati-1 (4.58 ± 0.0199) (Table 2). Further, the level of lipid peroxidation (μmoles g FW ^-1^) was determined through the estimation of MDA equivalents content in plants. The level of MDA was found to be more in sensitive cultivars IR64 (33.831 ± 0.058) and Pusa Basmati-1 (33.945 ± 0.047) than the tolerant cultivars Luna Sankhi (31.502 ± 0.206) and Luna Suvarna (29.414 ± 0.222) (Table 2).

**Table 2:** Comparative analysis of total protein, proline and MDA of 14 days old seedlings of different rice cultivars

Cultivar	Protein content (mg FW^-1^)	Proline content (μmoles g FW^-1^)	MDA content (μmoles g FW^-1^)
IR64	12.75 ± 0.275^b^	4.29 ± 0.0177^c^	33.831 ± 0.058^a^
PB	12.85 ± 0.352^b^	4.58 ± 0.0199^b^	33.945 ± 0.047^a^
Luna Sankhi	14.73 ± 0.275^a^	6.90 ± 0.0513^a^	31.502 ± 0.206^b^
Luna Suvarna	15.22 ± 0.403^a^	6.78 ± 0.2450^a^	29.414 ± 0.222^c^

Values represent mean ± SE (*n* = 3)

Different letters (^a^, ^b^, ^c^) within cultivars are significantly different (Tukey LSD, *p* ≤ 0.05)

### Chlorophyll content analysis

**Fig. 5:**
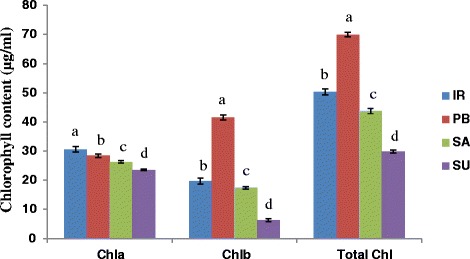
Comparative analysis of chlorophyll a, chlorophyll b and total chlorophyll in 14 days old seedlings of different rice cultivars. Bars represent mean ± SE (*n* = 3). Different letters (a, b, c, d) within cultivars are significantly different (Tukey LSD, *p* ≤ 0.05). (Chl: Chlorophyll, IR: IR64, PB: Pusa Basmati-1, SA: Luna Sankhi, SU: Luna Suvarna)

A significant difference in the content of chlorophyll a, chlorophyll b and total chlorophyll was observed in sensitive and tolerant cultivars of rice (Fig. 5). The maximum content of chlorophyll a (μg/ml) was found in IR64 (30.598 ± 0.076) followed by Pusa Basmati-1(28.418 ± 0.995) that was significantly higher from the Luna Sankhi (26.334 ± 0.128) and Luna Suvarna (23.569 ± 0.195). However, the highest content of chlorophyll b (41.537 ± 0.868) and total chlorophyll (69.931 ± 0.772) was found in Pusa Basmati-1 whereas the minimum content of these pigments was found in Luna Suvarana.

## Discussion

In the present study, it was observed that the tolerant cultivars (Luna Sankhi and Luna Suvarna) maintain a higher level of H_2_O_2_ and lower level of O^2-^ as compared to the sensitive cultivars (IR64 and Pusa Basmati-1). H_2_O_2_ acts as a secondary messenger that activates the various stress adaptive signalling pathways at both genetic and physiological levels [14]. It is known to activate various genes involved in the acclimation and tolerance to salt stress [15]. Further, enhanced H_2_O_2_ production in halophytes is essential for their “salt stress preparedness” [16]. Our study indicates that salt tolerant cultivars Luna Sankhi and Luna Suvarna are capable of constitutive activation of plant defence pathways due to the higher basal level of H_2_O_2_ that in turn keeps the tolerant cultivars ready for adaptation to salt stress conditions. Hence, H_2_O_2_ ‘signatures’ may be operating in salt stress signalling pathways in rice that keeps the defence pathway networks activated in tolerant cultivars [17]. Furthermore, the elevated basal level of H_2_O_2_ found in the tolerant cultivars could be attributed to the higher activity of NADPH oxidase and SOD enzyme in Luna Sankhi and Luna Suvarna that was further confirmed through the estimation of specific enzymatic activity of SOD. These findings suggest that the higher generation of O^2-^ in a NADPH dependent manner and its rapid conversion into signalling molecule H_2_O_2_ could be considered as a critical clue in understanding the model depicting better adaptation of tolerant cultivars towards salinity.

Once the signalling cascade has been initiated, plants are defended to the toxic effects of H_2_O_2_ with the help of APX, CAT and other peroxidases in a way similar to the Ca^2+^ efflux system that operates in well-known cytosolic calcium ‘signatures’ [6]. Similarly, in the present work, the specific activity of peroxidases APX and GPX was found to be high in tolerant varieties which could be scavenging the excess of H_2_O_2_ to prevent the plant from its ill effects, whereas the specific activity of CAT was found to be low. The less expression of CAT in tolerant cultivars could be due to the common substrate of APX and CAT and because of the higher affinity of APX for hydrogen peroxide than CAT [18]. Moreover, APX regulates the role of H_2_O_2_ as a signalling molecule unlike CAT which is more involved in detoxification [15].

Till now, the majority of research on the relation between ROS and salinity tolerance has been focussed on the enzymatic antioxidants although non-enzymatic antioxidants also play crucial role in scavenging excess of ROS and conferring tolerance [6]. Ascorbate is a known non enzymatic antioxidant that plays a central role in the ascorbate–glutathione cycle [19]. APX uses ascorbate as a reductant for the scavenging of H_2_O_2_ and converts it into dehydroascorbate. But for the maintenance of antioxidative capacity of ascorbate, its rapid regeneration into reduced form is necessary. APX functions in combination with GR, DHAR and MDHAR in glutathione-ascorbate pathway for the regeneration of ascorbate. In our studies, the specific activities of GR, DHAR and MDHAR enzymes were also found to be high in tolerant cultivars. Further the ratio of reduced to oxidised ascorbate was also observed to be high in Luna Sankhi and Luna Suvarna. This could be implied to the better operation of non-enzymatic glutathione-ascorbate pathway in tolerant cultivars which may be one of the many reasons for their superior salt tolerance ability [19]. Furthermore, the higher activity of various antioxidant enzymes (SOD, CAT, GPX, APX, and GR) in a coordinated manner in salt tolerant cultivars suggests that they are the major determinants in the model for depicting salt tolerance.

To further understand the dynamic role of antioxidant enzymes in conferring salt tolerance to halophytic varieties of rice, their transcript level expression study was conducted. Among the different isoforms of *SOD*, the expression of *Fe-SOD* and *Cu/Zn-SOD* was found to be high in tolerant cultivars whereas, the expression of *Mn-SOD* was more in sensitive cultivars. In an earlier study from our laboratory, we observed a higher expression of *Fe-SOD* and *Cu/Zn-SOD* in response to salt treatment in Pusa Basmati-1 [20]. Therefore, the higher basal expression level of *Fe-SOD* and *Cu/Zn-SOD* in tolerant cultivars could be one of the many reasons for their better adaptation in salt stress.

Furthermore, to get a better insight into the mechanism conferring elevated adaptation to salinity stress in Luna Sankhi and Luna Suvarna, some of the biochemical stress markers were studied. In the present study, enhanced level of total protein in Luna Sankhi and Luna Suvarna was observed which could be attributed to the synthesis of new or elevated level of proteins linked to stress tolerance in these cultivars. This result is further strengthened by the report that enhanced synthesis level of proteins is one of the major determinants of stress adaptation in halophytes [21]. Proline is a known osmoprotectant that also plays multiple antioxidant roles. Hyperaccumulation of free proline in plants is known in conferring salinity tolerance [22, 23]. The higher accumulation of free proline in Luna Sankhi and Luna Suvarna may be playing an important role in quenching of excess of ROS and stabilization of ROS-scavenging enzymes along with its well known role in osmoticum maintainence [24]. Further, with regard to reduced MDA content in tolerant cultivars, our results are in agreement to the previous studies where it has been shown that higher antioxidant enzyme activities are responsible for low MDA content which in turn inhibits the membrane damage by ROS and hence confers tolerance [25, 26]. It was interesting to note that the content of chlorophyll a, chlorophyll b and total chlorophyll was less in the tolerant cultivars in comparison to sensitive cultivars. Under salt stress conditions, plants are often observed to have reduced chlorophyll content [20, 27]. However, analysis of chlorophyll content in salt tolerant and sensitive cultivars will provide the physiological explanation to adaptation of plant to salinity stress and hence it is a subject of worth investigation. In our investigation the less chlorophyll contents in tolerant cultivars may be either due to the ROS mediated degradation of cholorophyll or it could be photocatalytic activity of cholorophyll itself for the production of H_2_O_2_ [28, 29]. In our case, we presume that in tolerant cultivars the salt tolerance could be attributed to the enhanced production of H_2_O_2_, which may be due to the photocatalytic activity of chlorophyll [30]. Moreover, the role of light in chlorophyll degradation has also been suggested [31].

## Conclusion

The tolerant cultivars of rice maintain a high threshold level of H_2_O_2_ due to more enzymatic activity of NADPH oxidase and enough SOD “in stock” that may constitutively activates the defence pathways resulting in higher intrinsic activity of various antioxidant enzymes. This provides certain adaptive advantage to halophytes over glycophytes because of which salt stress does not lead to oxidative stress in tolerant cultivars. Further, the first time comparative analysis of ROS generating system NADPH oxidase in halophytes and glycophytes provide a critical clue on understanding the model depicting salt tolerance in rice. We propose that these two systems work together in synchronization with each other for achieving salt tolerance. It will be interesting to further explore the crosstalk that exists between ROS generating and scavenging system for achieving salinity tolerance and whether this crosstalk operates in feedforward or feedback manner. Furthermore, the observation of degradation of chlorophyll for producing higher level of H_2_O_2_ and in turn offering tolerance points out towards an uncharted mechanism in salt stress adaptation biology.

## Methods

### Plant material

Four rice cultivars differing in salt stress tolerance level were selected for this work. Seeds of two salt sensitive cultivars viz. IR64 and Pusa Basmati-1 were procured from IARI (Indian Agricultural Research Institute, New Delhi) and the seeds of two salt tolerant cultivars Luna Sankhi and Luna Suvarna were collected from CRRI (Central Rice Research Institute Cuttack, Odisha, India). Luna Sankhi (CR Dhan 405) and Luna Suvarna (CR Dhan 403) are salt tolerant varieties of Indian origin that can tolerate salinity stress in the range of 6-8 dS m^-1^ [31].

### Surface sterilization and inoculation

Surface sterilization of the seeds was carried out according to Sharma’s method [26]. Healthy and mature seeds were selected and were sterilized using sodium hypochlorite and mercuric chloride. Sterilized seeds were then inoculated in autoclaved sand moistened with autoclaved double distilled water in plastic boxes and were kept at 25 ± 2 °C temperature with 14 h photoperiod under light intensity of 2000 lux for 14 days.

### Histochemical ROS detection

The histochemical level of different types of ROS was detected using three different dyes. For the detection of O^2-^, the second leaf of 14 d seedlings was cut into small pieces and these pieces were vacuum infiltered in 6 mM NBT prepared in 10 mM of sodium citrate buffer (pH = 6) for 10 min. They were incubated for 2 h at room temperature and the chlorophyll content of these pieces was then removed by boiling them in absolute ethanol till the complete removal of chlorophyll. After the removal of chlorophyll, pieces were observed under the stereo-microscope [32].

For the detection of H_2_O_2_, two different stains were used, DAB and H_2_DCFDA. For the DAB assay, small pieces of second leaf of 14 d seedlings were vacuum infiltered in DAB (1 mg/ml in water) for 10 min and were then incubated for 2 h under the dark conditions. Chlorophyll content was removed and they were observed under the stereo-microscope [32]. For H_2_DCFDA assay, pieces of second leaf were incubated in 10 μM H_2_DCFDA and were vacuum infiltered for 5 min. The leaves were then washed with double distilled water and were observed under the confocal microscope using laser beam of excitation 488 nm [33, 34].

### NADPH oxidase assay

#### Isolation of plasma membrane proteins

Plasma membrane proteins were extracted from the 0.5 g of fresh tissue [13]. The plantlets were homogenized in protein extraction buffer containing 0.25 M sucrose, 50 mM HEPES (pH 7.2), 3 mM ethylenediaminetetraacetic acid (EDTA), 1 mM dithiothreitol (DTT), 3.6 mM L-cysteine, 0.1 mM MgCl_2_, 0.6 % PVP and PIC (Protease Inhibitory Cocktail). The homogenate was filtered through two layers of muslin cloth and was centrifuged at 10,000 g for 45 min at 4 °C. Supernatant was ultracentrifuged at 1, 80, 000 g for 60 min at 4^0^ C. The pellet was resuspended in 10 mM chilled Tris-HCl. The protein content was determined according to method described by Bradford [35] using Bovine Serum Albumin (BSA) as a standard.

#### Spectrophotometric assay of NADPH oxidase activity

The O^2-^ generating activity of NADPH oxidase was measured by following the Kaundal’s method [13]. The assay mixture was prepared with 50 mM Tris-HCl buffer (pH 7.5), 1 mM XTT (sodium, 3-[1-[phenylamino-carbonyl]-3, 4-tetrazolium]-bis(4-methoxy-6-nitro)benzenesulfonic acid hydrate), 1 mM NADPH, and 20 μg of membrane protein. The rate of reduction of XTT by O_2_^-^ was determined spectrophotometrically at 492 nm using an extinction coefficient of 2.16 x 10^4^ M^-1^ cm^-1^.

### Estimation of aqueous H_2_O_2_ content

The content of aqueous H_2_O_2_ was estimated using the modified ferrous oxidation-xylenol orange (FOX) assay [13]. 0.5 g of fresh seedlings of 14 d plants was homogenised in activated charcoal (0.1 g) prepared in 5 ml of 5 % trichloroacetic acid. Homogenate was filtered through Whatmann filter No.1 and was centrifuged at 8000 rpm for 10 min. The supernatant was used for the estimation of aqueous H_2_O_2_. FOX reagent was prepared using 1 ml of reagent ‘a’ (25 mM ammonium ferrous sulfate prepared in 2.5 M sulfuric acid), 50 μl of reagent ‘b’ (0.25 M xylenol orange prepared in HPLC-grade methanol), and 90 ml of reagent ‘c’ (9.69 mg of butylated hydroxytoluene prepared in 90 ml of HPLC- grade methanol). The total volume was raised to 100 ml with distilled water. In a test tube, 0.2 ml of plant extract was taken and 1 ml FOX reagent was added to it. The reaction mix was mixed properly and was incubated at room temperature for 15 min. All solutions were prepared fresh and used within 2 h. The content of aqueous H_2_O_2_ was estimated by recording the absorbance at 560 nm. Concentration was calculated using a standard curve prepared by taking varying concentrations of H_2_O_2._

### Extraction of protein

For the estimation of antioxidant enzyme activities, 1 g of fresh 14 d seedlings were homogenised in 3 ml of chilled buffer containing 50 mM phosphate buffer (pH = 7.8), 2 mM EDTA, 1 mM DTT, 1 mM PMSF (Phenylmethylsulfonyl Fluoride), 0.5 %(v/v) Triton X-100 and 10 % (w/v) PVP-40 (Polyvinylpyrrolidone). The homogenate was centrifuged for 20 min at 12,000 rpm and the supernatant was collected. This supernatant was then used for various enzymatic assays [26]. Protein concentration was determined using Bradford assay with BSA as a standard [35].

### Estimation of antioxidant enzyme activities

The enzymatic activities of different antioxidant enzymes were determined spectrophotometrically. The EC (Enzyme Commission) number mentioned against each enzyme is a unique number in the numerical classification scheme for enzymes and represent the chemical reactions they catalyze.

- SOD assay (EC 1.15.1.1)

The enzymatic activity of SOD was determined using the method given by Beauchamp and Fridovich [36]. 1 ml of reaction mixture was prepared in 50 mM potassium phosphate buffer using 2 μM riboflavin, 75 μM Nitrotetrazolium blue (NBT), 100 μM EDTA, 13 mM DL-methionine and 50 μl of enzyme extract and the absorbance was taken at 560 nm. The reaction mix was illuminated for 20 min at 25 °C for initiation. 1 unit of enzyme activity is expressed as the amount of enzymes required for 50 % inhibition of NBT reduction at 25 °C.

b) CAT assay (EC 1.11.1.6)

CAT specific activity was estimated at 25 °C using the method given by Aebi [37]. Decrease in absorbance of H_2_O_2_ was measured in 1 ml of reaction mixture having 10 mM H_2_O_2_ and 20 μl of enzyme extract in 50 mM of potassium phosphate buffer (pH = 7). Specific enzyme activity was expressed as l mole of H_2_O_2_ decomposed mg protein^-1^ min^-1^.

c) APX assay (EC 1.11.1.11)

The specific activity of APX was assayed as the rate of oxidation of ascorbate in the presence of H_2_O_2_ [26]. The reaction mixture of 1 ml comprised of 0.5 mM ascorbate, 0.1 mM H_2_O_2_ and 0.1 mM EDTA, potassium phosphate buffer(pH = 7) and 10 μl of enzyme extract. Decrease in absorption was observed spectrophotometerically at 290 nm at 25 °C. One unit of enzyme activity was expressed as the amount of enzyme required to oxidise 1 μM of ascorbate/minute/g tissue.

d) GR assay (EC 1.6.4.2)

The specific activity of GR is determined by analysing the decrease in absorbance at 340 nm [26]. 1 ml reaction mixture used for the assay had 50 mM potassium phosphate buffer (pH = 7.8), 1 mM EDTA, 1 mM oxidized glutathione (GSSG) and 25 μl enzyme extract. The reaction was started by adding 0.1 mM NADPH at last. Enzyme activity was expressed as μmol of NADPH oxidised min^-1^ mg protein^-1^.

e) GPX assay (EC 1.11.1.7)

The activity of GPX is observed by measuring the increase in absorbance at 436 nm [26]. The reaction mixture was prepared in 50 mM potassium phosphate buffer (pH = 7) having 9 mM guaiacol, 10 mM H_2_O_2_ and 33 μl of enzyme extract. The enzymatic activity of GPX is expressed as the amount of enzyme required to produce 1 μmol guaiacol dehydrogenation product min^-1^ mg protein^-1^.

f) DHAR assay (EC 1.8.5.1)

The specific activity of DHAR was estimated by measuring the increase in absorbance at 25 °C due to the formation of ascorbate from dehydroascorbate at 265 nm [26]. The reaction mixture of 1 ml consisted of 50 mM of potassium phosphate buffer (pH = 7), 0.1 mM EDTA, 0.5 mM dehydroascorbate, 2.5 mM GSH and 25 μl of enzyme extract. One unit of enzyme activity was expressed as amount of enzyme required to produce 1 μmol of ascorbate min^-1^ mg protein^-1^.

g) MDHAR assay (EC 1.6.5.4)

The specific activity of MDHAR was determined at 25 °C by measuring the increase in absorbance at 340 nm [26]. The 1 ml reaction mixture used for this estimation consisted of 50 mM Tris-HCl buffer (pH = 7.6), 0.15 units of ascorbate oxidase enzyme, 2.5 mM ascorbic acid and 0.2 mM NADPH/NADH. One unit of enzyme activity was given as the amount of enzyme required to oxidase 1 μmol of NADPH min^-1^ mg protein^-1^.

### Expression analysis of antioxidant enzymes

For the gene expression studies of various antioxidant genes, semi quantitative primers were designed using the IDT software (www.idtdna.com) (Table 3). Total RNA from the 14 d plants was extracted using the trizol method as per the manufacturer’s instructions. A total of 3 μg of RNA was used for the cDNA preparation with commercial cDNA synthesis kit. Gene expression was studied in 50 μl of polymerase chain reaction (PCR) using 50 ng of cDNA template. The PCR parameters used were: predenaturation at 94 °C for 4 min, followed by 35 cycles of 94 °C for 1 min, 55 °C for 1 min, 72 °C for 1 min, with a final extension step of 72 °C for 7 min. Rice elongation factor *ef1α* was used as internal control. All the PCR’s were performed at least with three independent samples and intensity of the products was confirmed with 1 % agarose gel with ethidium bromide. The relative level of transcript in each PCR reaction was determined using the integerated density value (IDV), with Alpha 2000TM Image Analyzer software.

**Table 3:** List of primers for RT-PCR

S. No.	Name of the gene	Database	Accession number	Primer sequence
1	*EF1α*	GenBank	D63580.1	F: 5^0^-GTACAAGATCGGTGGTATT-3^0^
				R: 5^0^-GGGTACTCAGAGAAGGTCT-3^0^
2	Cu/Zn-SOD	GenBank	L19435.1	F: 5^0^-CCTCAAGCCTGGTCTCCAT-3^0^
				R:5^0-^CAGCCTTGAAGTCCGATGAT-3^0^
3	Fe-SOD	GenBank	AY770495.1	F: 5^0^-CTTGATGCCCTGGAACCTTA-3^0^
				R: 5^0^-GCCAGACCCCAAAAGTGATA-3^0^
4	Mn-SOD	GenBank	L19436.1	F: 5^0^-GCCATTGATGAGGATTTTGG-3^0^
				R: 5^0^-CAAGCAGTCGCATTTTCGTA-3^0^
5	CAT	GenBank	D26484.1	F: 5^0^-GTTCGGTTCTCCACAGTCGT-3^0^
				R: 5^0^-CCCTCCATGTGCCTGTAGTT-3^0^
6	APX	GenBank	D45423.1	F: 5^0^-CCAAGGGTTCTGACCACCTA-3^0^
				R: 5^0-^CAGTTCGGAGAGCTTGAGGT-3^0^
7	GR	GenBank	AB009592.1	F: 5^0-^AACAGCCGATGGCATAAAAG-3^0^
				R:5^0^-CAACCACCAGTTTCATGACG-3^0^

### In-gel SOD assay

For in-gel SOD assay, 100 mg of fresh seedling tissue of each cultivar was homogenized with 50 mM Tris HCl buffer (pH = 7.5). The homogenate was centrifuged at 12000 rpm for 15 min at 4 °C [38]. The protein content was quantified in the supernatant using Bradford assay with BSA as a standard. Native-PAGE analysis was carried out with 50 μg protein of each cultivar at 80 V stacking and 100 V resolving. The gel was then stained according to Rucinska’s method using 2.45 mM NBT prepared in 50 mM potassium phosphate buffer for 20 min in dark [39]. The gel was again washed with autoclaved water followed by immersion in potassium phosphate (50 mM, pH 7.8) containing TEMED (28 mM) and riboflavin (3 μM) for 15 min. The gel was then kept on dry white illumination tray till the gel became uniformly blue except at positions containing SOD and maximum contrast between the achromatic zones and general blue colour was achieved.

### Ascorbate content

The content of ascorbic acid was estimated using the Dutilleul’s method with minor modifications [40]. 1 g of fresh leaf tissues of four cultivars was homogenized with 3 % metaphosphoric acid (3 %) containing EDTA (1 mM). The homogenate was centrifuged at 8000 × g for 15 min. 0.1 ml of supernatant was taken and was mixed with 1 ml citrate phosphate buffer (0.1 M, pH 2.1). The absorbance was taken at 265 nm and 1 unit of APX was added. The solution was incubated for 5 min at room temperature and absorbance was again noted at 265 nm. The difference in two readings of absorbance was calculated that indicated the reduced ascorbate content utilized specifically by APX. For the estimation of total ascorbate content, 0.1 ml of DTT (0.1 M) was added in another set and absorbance was read at 265 nm after 5 min. 1 unit of APX was added and absorbance was taken after 5 min. For the determination of oxidized ascorbate content, reduced ascorbate content was subtracted from the total ascorbate content. Calibration curve was prepared using graded concentration of L-ascorbic acid in 3 % HPO_3_. The ratio of reduced ascorbate and oxidized ascorbate was also determined.

### Proline content

Proline content was determined using the method given by Bates [41]. 0.5 g of total seedlings were crushed in liquid nitrogen and homogenised in 3 % aqueous sulfosalicyclic acid. The samples were then centrifuged at 12000 rpm for 15 min and 2 ml of the supernatant was taken in a test tube. Equal amount of acid ninhydin and glacial acetic acid was added to the supernatant and the reaction mix was kept in boiling water for 1 h. The reaction was stopped by keeping the tubes on ice. Added 4 ml of toluene to the reaction mixture and toluene layer separated from the aqueous phase was collected. The proline content was determined by measuring the absorbance of the toluene at 520 nm. The amount of proline was estimated using the proline standard curve and was expressed as μmoles gFW^-1^.

### Lipid peroxidation

The level of lipid peroxidation was determined using the TBARS assay [42]. 1 g of tissue sample was homogenised in 3 ml of 0.1 % of chilled trichloroacetic acid (TCA) and 3 ml of solution containing 0.5 % TBA (Thiobarbituric acid) in 20 % TCA was added to it. The reaction mixture was incubated at 95 °C for 30 min and the reaction was stopped by keeping the tube on ice. Centrifuged the mixture at 10,000 rpm for 15 min. The supernatant was collected and its absorbance was measured at 532 nm, with a reading at 600 nm subtracted from it to account for nonspecific turbidity. 155 mM^-1^cm^-1^ extinction coefficient was used for the quantification of MDA-TBA complex and was expressed as μmoles gFW^-1^.

### Chlorophyll content

Chlorophyll content was estimated using the method given by Arnon with some modifications [43]. 100 mg of fresh leaves of each cultivar were taken and were homogenised in liquid nitrogen. To the homogenised tissue sample, 1.5 ml of 80 % acetone was added and the reaction was incubated in dark for 1 h. Samples were centrifuged at 15,000 rpm for 3 min. The supernatant was collected and the absorbance was measured spectrophotometrically at 645 and 663 nm against 80 % acetone as blank. The chlorophyll content was determined as follows:

$$ \mathrm{Total}\ \mathrm{chlorophyll}\ \left(\upmu \mathrm{g}/\mathrm{ml}\right)\kern0.5em =\kern0.5em 20.2\ \left({\mathrm{A}}_{645}\right)\kern0.5em +\kern0.5em 8.02\left({\mathrm{A}}_{663}\right) $$

$$ \mathrm{Chlorophyll}\ \mathrm{a}\ \left(\upmu \mathrm{g}/\mathrm{ml}\right)\kern0.5em =\kern0.5em 12.7\kern0.5em \left({\mathrm{A}}_{663}\right)-2.69\left({\mathrm{A}}_{645}\right) $$

$$ \mathrm{Chlorophyll}\ \mathrm{b}\kern0.5em \left(\upmu \mathrm{g}/\mathrm{ml}\right)\kern0.5em =\kern0.5em 22.9\kern0.5em \left({\mathrm{A}}_{645}\right)-4.68\left({\mathrm{A}}_{663}\right) $$

### Statistical analysis

Data from different experiments was analysed statistically with one-way analysis of variance (ANOVA). The value of different parameters is expressed as mean ± SE of three independent replicates. The Tukey LSD test was applied for multiple comparisons using Sigma stat version 3.5, and significance of difference between the cultivars were set as *p* ≤ 0.05.

## Abbreviations

APX, ascorbate peroxidase; CAT, catalase; DAB, 3, 3-diaminobenzidine; DHAR, dehydroascorbate reductase; GPX, guaicol peroxidase; GR, glutathione reductase; H_2_DCFDA, 2',7'-dichlorodihydrofluorescein diacetate; H_2_O_2_, hydrogen peroxide; MDA, malondialdehyde; MDHAR, monodehydroascorbate reductase; NADPH, nicotinamide adenine dinucleotide phosphate hydrogen; NBT, nitrozolium blue; O^2-^, superoxide ions; PCR, polymerase chain reaction; PIC, protease inhibitory cocktail; PMSF, phenylmethylsulfonyl fluoride; ROS, reactive oxygen species; semi-RT, semi quantitative reverse transcriptase; SOD, superoxide dismutase; XTT, sodium3-[1-[phenylamino-carbonyl]-3,4-tetrazolium]-bis(4-methoxy-6-nitro)benzenesulfonic acid hydrate
